# An LRP5 Receptor with Internal Deletion in Hyperparathyroid Tumors with Implications for Deregulated WNT/β-Catenin Signaling

**DOI:** 10.1371/journal.pmed.0040328

**Published:** 2007-11-27

**Authors:** Peyman Björklund, Göran Åkerström, Gunnar Westin

**Affiliations:** Department of Surgical Sciences, Uppsala University, Endocrine Unit, Uppsala University Hospital, Uppsala, Sweden; Utrecht University, The Netherlands

## Abstract

**Background:**

Hyperparathyroidism (HPT) is a common endocrine disorder with incompletely understood etiology, characterized by enlarged hyperactive parathyroid glands and increased serum concentrations of parathyroid hormone and ionized calcium. We have recently reported activation of the Wnt signaling pathway by accumulation of β-catenin in all analyzed parathyroid tumors from patients with primary HPT (pHPT) and in hyperplastic parathyroid glands from patients with uremia secondary to HPT (sHPT). Mechanisms that may account for this activation have not been identified, except for a few cases of β-catenin (*CTNNB1*) stabilizing mutation in pHPT tumors.

**Methods and Findings:**

Reverse transcription PCR and Western blot analysis showed expression of an aberrantly spliced internally truncated WNT coreceptor low-density lipoprotein receptor–related protein 5 (LRP5) in 32 out of 37 pHPT tumors (86%) and 20 out of 20 sHPT tumors (100%). Stabilizing mutation of *CTNNB1* and expression of the internally truncated LRP5 receptor was mutually exclusive. Expression of the truncated LRP5 receptor was required to maintain the nonphosphorylated active β-catenin level, transcription activity of β-catenin, *MYC* expression, parathyroid cell growth in vitro, and parathyroid tumor growth in a xenograft severe combined immunodeficiency (SCID) mouse model. WNT3 ligand and the internally truncated LRP5 receptor strongly activated transcription, and the internally truncated LRP5 receptor was insensitive to inhibition by DKK1.

**Conclusions:**

The internally truncated LRP5 receptor is strongly implicated in deregulated activation of the WNT/β-catenin signaling pathway in hyperparathyroid tumors, and presents a potential target for therapeutic intervention.

## Abstract 2

Gunnar Westin and colleagues report the expression of an aberrantly spliced LRP5 receptor in primary and spontaneous parathyroid tumors and implicate it in the deregulated activation of the Wnt/β-catenin signaling pathway.

## Editors' Summary

**Background.:**

The parathyroid glands—four rice-sized glands in the neck—maintain a normal calcium balance in the body, to maintain strong bones and essential cellular functions. The glands release parathyroid hormone as a response to a decrease in blood calcium level. By stimulating calcium release from bone and its absorption in the gut, parathyroid hormone restores the blood calcium level. However, 100,000 new individuals in the US develop hyperparathyroidism (HPT) annually, characterized by enlarged, overactive parathyroid glands and high blood levels of calcium. Primary HPT (pHPT) is usually caused by a benign tumor (a non-life-threatening growth) in one of the parathyroid glands. Secondary HPT (sHPT) occurs in response to calcium regulatory disturbances, linked to vitamin D deficiency, and more or less invariably develops in patients with uremic kidney disease.

**Why Was This Study Done?:**

HPT is usually treated by surgical removal of the enlarged parathyroid glands, which is done with great efficiency. However, ideally, doctors would like to know what drives the overgrowth of the parathyroid glands to be able to develop drugs for treatment or disease prophylaxis. Researchers recently reported that the cells in enlarged parathyroid glands from patients with HPT contain high amounts of β-catenin. This protein is part of the Wnt signaling pathway, which has been found to be disrupted in many tumor entities in other organs. In the absence of Wnt proteins, a group of proteins called the β-catenin destruction complex marks β-catenin so that it is rapidly destroyed. When Wnt proteins bind to a cell-surface receptor called Frizzled and a coreceptor called LRP5, the destruction complex is inhibited and β-catenin accumulates. This accumulation induces the production of other proteins (in particular, c-Myc) that stimulate cell growth and division. The accumulation of β-catenin in the enlarged parathyroid glands of patients with HPT could, therefore, significantly contribute to the overgrowth of their glands—but what causes β-catenin accumulation? In this study, the researchers have investigated this question to try to identify a target for drugs to treat HPT.

**What Did the Researchers Do and Find?:**

The researchers looked for genetic changes (mutations) in β-catenin that stabilize the protein and measured the expression of LRP5 in abnormal parathyroid gland tissue from 37 patients with pHPT and 20 with uremia and sHPT. All the samples contained high levels of β-catenin, but only four contained a β-catenin–stabilizing mutation. All the sHPT samples and 32 pHPT samples (but none of the samples containing the β-catenin stabilizing mutation) expressed a mutated LRP5, with the central region deleted. To investigate the functional consequences of this internally deleted LRP5 protein, the researchers used a technique called RNA interference to block its expression in a human parathyroid tumor cell line. They found that expression of the mutated, short LRP5 is required for accumulation of β-catenin, expression of c-Myc, and continued growth of the cell line in test tubes and in animals.

**What Do These Findings Mean?:**

The accumulation of β-catenin in all the enlarged parathyroid glands examined so far strongly implicates abnormal Wnt/β-catenin signaling in the development of pHPT and sHPT. These new findings identify which part of the signaling pathway is altered. The expression data and functional data together suggest that an internally deleted LRP5 coreceptor is often responsible for the accumulation of β-catenin. The functional data also show that expression of shortened LRP5 is necessary for the abnormal growth of parathyroid tumor cells. Exactly how the internally deleted coreceptor activates β-catenin signaling in parathyroid gland cells, or why a shorter-than-normal LRP5 is made, are not yet known. However, because these findings indicate that internally deleted LRP5 has a fundamental role in activating Wnt signaling in HPT, drugs that inactivate this aberrant protein but leave the normal protein unscathed might provide a nonsurgical treatment for this common hormone disorder.

**Additional Information.:**

Please access these Web sites via the online version of this summary at http://dx.doi.org/10.1371/journal.pmed.0040328.

- edlinePlus has encyclopedia pages on hyperparathyroidism, primary hyperparathyroidism, and secondary hyperparathyroidim (in English and Spanish)
- Information is available for patients from the US National Institute of Diabetes and Digestive and Kidney Diseases on hyperparathyroidism, which includes links to organizations that help people with hyperparathyroidism
- Wikipedia maintains pages on Wnt signaling pathway and on β-catenin (note that Wikipedia is a free online encyclopedia that anyone can edit; available in several languages)

## Introduction

Primary hyperparathyroidism (pHPT) is characterized by hypersecretion of parathyroid hormone and generally also hypercalcemia, due to one or several parathyroid tumors (adenoma). Secondary hyperparathyroidism (sHPT) develops in patients with uremia because of phosphate retention, hypocalcemia, and reduced 1,25-dihydroxyvitamin D_3_ levels, causing parathyroid hyperplasia and eventually development of parathyroid tumors and hypercalcemia [1–4]. Parathyroidectomy is the only considered therapy for most patients. We recently reported aberrant β-catenin (CTNNB1) accumulation in all analyzed parathyroid tumors from patients with pHPT and in hyperplastic parathyroid glands from patients with uremia secondary to HPT [5]. *MYC*, a direct target of the Wnt/β-catenin signaling pathway in colorectal cancer cells and established as the critical mediator of the early stages of intestinal neoplasia [6,7], was found to be overexpressed at the protein level in 79% of parathyroid tumors [5]. Maintained activity of endogenous β-catenin was found to be necessary for the expression of *MYC* and cyclin D1 (*CCND1*), as well as growth and survival of a unique human parathyroid tumor cell line [8]. Overexpression of cyclin D1 has been reported in 20%–40% of pHPT tumors [2], and overexpression of cyclin D1 in the parathyroid glands of transgenic mice caused development of pHPT [9]. In a small fraction of parathyroid adenomas, overexpression is due to activation of the *CCND1* gene by pericentromeric inversions of Chromosome 11, involving the parathyroid hormone (*PTH*) promoter [10]. Augmented cyclin D1 expression in some parathyroid adenomas could also be a consequence of aberrant β-catenin accumulation [5], although it remains to be determined whether *CCND1* constitutes a β-catenin target [11] in parathyroid cells. We also reported *CTNNB1* stabilizing mutations in a few cases (3 out of 20) of pHPT tumors, while no mutation was found in uremic secondary HPT tumors, and inactivating truncations of adenomatosis polyposis coli (APC) were not seen [5]. Mutation or deregulated expression of other Wnt-signaling components leading to β-catenin accumulation was therefore anticipated.

Dysregulated Wnt signaling with accumulation of β-catenin in the cytoplasm/nucleus plays an important role in a variety of human cancers. The stability of β-catenin is regulated by Wnt ligands through a “destruction complex” consisting of APC/Axin/GSK-3β/Ck1/Dvl and other factors. In the absence of Wnt ligand, free cytoplasmic β-catenin is rapidly degraded by the proteasome after phosphorylation of its amino terminus at residues serine 33, serine 37, threonine 41, and serine 45 [12–15]. Wnt ligands bind to cell-surface Frizzled receptors and LRP5/6 coreceptors and result in changes in phosphorylation of several intracellular signaling components with the subsequent accumulation of nonphosphorylated β-catenin [16–19]. According to a current model, the destruction complex is inactivated through recruitment of Axin to the intracellular domain of LRP5 [20]. β-catenin binds the LEF/TCF family of transcription factors to positively or negatively regulate transcription of target genes. Many mutant proteins of the Wnt signaling pathway, such as β-catenin, APC, Axin, and beta-transducin repeat-containing protein (β-Trcp), are associated with specific forms of cancer. For instance, aberrant accumulation of β-catenin through stabilizing mutations in *CTNNB1* or inactivating mutations in *APC* is strongly implicated in the cause of approximately 10% and 80% of colorectal cancers, respectively [13,14]. A mutant of LRP5 lacking the extracellular domain was demonstrated to be constitutively active in vitro [20]. In this study, we aimed at investigating the potential role of LRP5 in parathyroid tumorigenesis.

## Methods

### Tissue Specimens

Parathyroid adenomas (*n* = 37) and hyperplastic glands (*n* = 20) from patients with pHPT and sHPT, respectively, were acquired from patients diagnosed and operated on in the clinical routine. Each patient contributed with one tumor. All 57 tumors displayed aberrant accumulation of β-catenin (unpublished data), of which 14 parathyroid adenomas and all 20 hyperplastic parathyroid glands were described previously [5]. Normal parathyroid tissue (*n* = 6) was obtained from glands inadvertently removed in conjunction with thyroid surgery where autotransplantation was not required or as normal parathyroid gland biopsies in patients subjected to parathyroidectomy. All tissues were intraoperatively snap-frozen, and cryosections were used in the analyses. Written informed consent and approval of local ethics committee was obtained.

### Detection of Normal and Internally Truncated LRP5 Transcripts by PCR and DNA Sequencing

Total RNA was extracted with TriZol Reagent (Gibco BRL, Life Technologies) according to the manufacturer's instructions and the RNA was subsequently treated with RQ1 DNase I (Promega) and proteinase K. Alternatively, DNA-free RNA was prepared using the Nucleospin RNA II kit (Macherey-Nagel). Successful DNase treatments were established by PCR analysis of all RNA preparations. Reverse transcription of total DNA-free RNA was performed with random hexamer primers using the First-Strand cDNA Synthesis kit (Amersham Pharmacia Biotech) according to the manufacturer's instructions. cDNA was amplified by primary or nested PCR using mRNA-specific primers spanning positions 1992–2932 of LRP5 (GenBank accession number AF064548; http://www.ncbi.nlm.nih.gov/Genbank). A total of 1%–2% of the primary PCR product was used for nested PCR. Primers used were the following: forward primer, 5′-CTTCACCAGCAGAGCCGCCATCCACAG-3′; nested forward, 5′-GGATCTCCCTCGAGACCAATAACAACG-3′; and reverse, 5′-CCGGGATCATCC GACTGATG-3′. The PCR amplifications were performed with cDNA, 25 pmol of each primer, 0.2 mM dNTPs, 1× PCR buffer, 1.5 mM MgCl_2_, and 0.25 U Platinum Taq DNA polymerase (Invitrogen). The PCR conditions were: denaturation at 95 °C for 60 s, followed by 40 cycles of denaturation for 20 s, annealing at 58 °C for 20 s and extension at 72 °C for 90 s, and a final extension at 72 °C for 7 min. An annealing temperature of 61 °C and 40 cycles were used for nested amplification. Two LRP5wt and six randomly chosen truncated LRP5 cDNA fragments from parathyroid tumors, as well as from sHPT-1 cells, were cloned into pCRII-TOPO (Invitrogen), and sequenced on ABI 373A using the ABI Prism Dye Terminator Cycle Sequencing Ready Reaction kit (Applied Biosystems). All fragments encoded an open reading frame, and the seven truncated fragments contained the same in-frame deletion (Δ666–809). Normal tissue cDNA or RNA were purchased from BD Biosciences Clontech and Ambion.

### Cell Growth Determination and Flow Cytometry

Cells (2 × 10^5^) were distributed onto 35-mm dishes in DMEM/10% fetal bovine serum and subsequently harvested at the indicated time points. The number of viable cells were determined by using the NucleoCounter (ChemoMetec). Cells were collected at 84 h after siRNA transfection and incubated with FITC-labeled annexin V to assess phosphatidylserine externalization as a marker for apoptosis.

Propidium iodide was added to distinguish tumor cells that had lost membrane integrity. Cells were analyzed by flow cytometry on a Becton Dickinson FACS Calibur flow cytometer (BD Biosciences).

### RT-PCR Analyses

cDNA was prepared as described above. The following mRNA-specific PCR primers and labeled probes (5′FAM-sequence–3′TAMRA) were used for quantitative real-time RT-PCR analysis. For LRP5wt: forward: 5′-CCTGAAGACCATCAGCCGCG-3′; reverse, 5′-CCCGCTCCTGACCCAGCATG-3′; and probe, 5′-TCCCACCAAGGGCTACATCTACTG-3′. For LRP5tot: forward, 5′-ATCGACTGTATCCCCGGGGC-3′; reverse, 5′-CACCACGCGCTGGCACACAA-3′; and probe, 5′-CGGACTGTGACGCCATCTGCCTGC-3′. For MYC: forward, 5′-AAGACTCCAGCGCCTTCTCTCCGT-3′; reverse, 5′-TGGGCTGTGAGGAGGTTTGCTGTG-3′; and probe, 5′-AGCGACTCTGAGGAGGAACAAGAA-3′. For 28S rRNA, the Ribosomal RNA Control Reagents (VIC probe) was used (Applied Biosystems). PCR reactions were performed on ABI PRISM 7700 Sequence Detection System or MyiQ Single-Color Real-Time PCR Detection System (Bio-Rad) using the TaqMan PCR core Reagent Kit (Applied Biosystems). Each cDNA sample was analyzed in triplicate. Standard curves for the expressed genes were established by amplifying a purified PCR fragment covering the sites for probes and primers. Messenger RNA-specific PCR primers for WNT1 were as described [21]. Primers for WNT3 were as follows: forward, 5′-GGCTGTGACTCGCATCATAA-3′; reverse, 5′-CAGCAGGTCTTCACCTCACA-3′.

### Transfection Experiments, Western Blotting, and Immunoprecipitation

sHPT-1 parathyroid tumor cells [8] were transfected with siRNA at least in triplicates at 2 × 10^5^ cells/35-mm dish with jetSI-ENDO according to the manufacturer's recommendations (Poly-Plus-Transfection SAS). The following siRNAs were used: control nonsilencing siRNA (Qiagen Operon), siLRP5Δ666–809; 5′-TAACAACGACCUCACCAUUdTdT-3′ and 5′-AAUGGUGAGGUCGUUGUUAdTdT-3′ (synthesized by Thermo Electron, Ulm, Germany), siLRP5wt; 5′-CAACCACAUCUACUGGACAdTdT-3′ and 5′-UUCCAGUAGAUGUGGUUGdTdT-3′ (Thermo Electron), siLRP5tot; stealth 5′-CCUGCAUGGACUGAGGAACGUCAAA-3′ and stealth 5′-UUUGACGUUCCUCAGUCCAUGCAGG-3′, control siLRP5tot; stealth 5′-CCUGGUAGUCAAGGAUGCACCGAAA-3′ and stealth 5′-UUUCGGUGCAUCCUUGACUACCAGG-3′ (Invitrogen). A very high transfection efficiency (virtually all cells) was obtained for sHPT-1 cells by this protocol [8]. HeLa cells were transfected in the same manner, with a transfection efficiency of approximately 90% (data not shown). Conditioned medium was produced in HEK293T cells transiently transfected for 24 h by pCIN4/Wnt1 (kind gift from Dr. R. Kemler), pLNC Wnt-3HA (kind gift from Dr. J. Kitajewski), PON-Wnt-3a (kind gift from Dr. B. Williams), or pCS2/Dkk1 (kind gift from Dr. S. Y. Sokol) using Fugene 6 (Roche Diagnostics). sHPT-1 cells were transfected with the FOPFLASH or TOPFLASH TCF [22] luciferase reporter (Upstate) and the CMV-LacZ reference plasmid [23] for 24 h using jetPEI (Poly-Plus-Transfection SAS). In some experiments, TOPFLASH was transfected; transfection of siRNA to the same culture was done after 24 h and cells were harvested 96 h later. Luciferase and β-galactosidase activities were determined luminometrically, and luciferase activity was normalized for differences in β-galactosidase activity. Expression plasmid LRP5Δ666–809 was constructed by replacing the XhoI/KpnI fragment of pcDNA3.1/LRP5 and pcDNA3.1/V5-His/LRP5 (expressing tagged LRP5) with a XhoI/KpnI-digested PCR fragment harboring the deletion Δ666–809. Western blotting analyses were done on extracts prepared in Cytobuster Protein Extract Reagent (Novagen) supplemented with Complete protease inhibitor cocktail (Roche Diagnostics) or on a cytosolic protein extract [24]. Anti-V5–HRP antibody (Invitrogen), anti-active–β-catenin [25] mouse monoclonal antibody (Upstate; catalog number 05–665), anti-LRP5 goat polyclonal antibody (Santa Cruz Biotechnology; catalog number sc-21390), anti-actin goat polyclonal antibody, and anti–β-tubulin rabbit polyclonal antibody (Santa Cruz Biotechnology) were used. For tumor specimens, protein extracts for Western blotting were prepared from 10 consecutive frozen tissue sections (6 μm) in Cytobuster Protein Extract Reagent (Novagen) supplemented with Complete protease inhibitor cocktail (Roche Diagnostics). For immunoprecipitations, cells were resuspended in 300 μl buffer (50 mM Tris [pH 8.0], 150 mM Nacl, 0.5% NP-40, 50 mM NaF, and 1 mM EDTA, supplemented with Complete protease inhibitor cocktail), kept on ice for 20 min, and centrifuged for 20 s at 14,000 rpm. After addition of 20 μl anti-LRP5 goat polyclonal antibody (Santa Cruz Biotechnology; catalog number sc-21390), the lysate was incubated overnight at 4 °C with gentle agitation. A total of 50 μl Protein G PLUS–Agarose (Santa Cruz Biotechnology; catalog number sc-2002) was then added to the lysate and further incubated for 6 h. After centrifugation, the sample was boiled in 40 μl Laemmli sample buffer for 10 min and 10–20 μl was subjected to Western blotting analysis.

### ChIP Assay

Chromatin immunoprecipitation (ChIP) of transfected cells was performed using a protocol from Upstate, but with immunoprecipitation conditions as described [26]. The anti-active–β-catenin mouse monoclonal antibody [25] was used (Upstate; catalog number 05–665) and *MYC* promoter DNA, containing TCF-4 binding site 2 [22], was PCR amplified in the linear range by the following primers: forward, 5′-ACGTGGCAATGCGTTGCTGGG-3′; and reverse, 5′-ACACAGAGAACGCACTGCGCG-3′.

### Mouse Xenograft Model

Female Fox Chase severe combined immunodeficiency (SCID) mice (2- to 3-wk-old) were used (Taconic). The mice were anesthetized with isoflurane (Forene; Abbott ) during the manipulations. One flank or both flanks of each animal were injected subcutaneously (total 200 μl) with sHPT-1 cells together (1:1) with BD Matrigel Matrix (BD Biosciences Clontech) after transfection of 10^6^ cells for 24 h. The animals were monitored every day and humanely killed after 8–9 wk. The animal experiments were approved by the Uppsala University board of animal experimentation and were performed according to the United Kingdom Coordinating Committee on Cancer Research guidelines for the welfare of animals in experimental neoplasia [27].

### Statistical Analysis

Unpaired *t* test was used for all statistical analyses. Values are presented as arithmetrical mean ± standard error of the mean. A *p*-value below 0.05 was considered significant.

## Results

### An Internally Truncated LRP5 Receptor Is Expressed in Parathyroid Tumors

**Figure 1:**
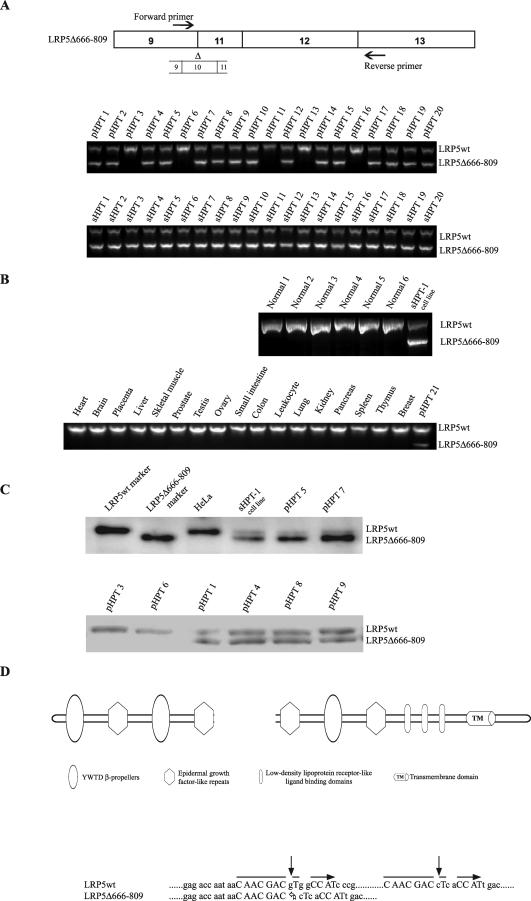
An Internally Truncated LRP5 Receptor Is Expressed in Parathyroid Tumors (A) RT-PCR analysis of RNA from pHPT tumors (*n* = 20) and sHPT tumors (*n* = 20) using primers located in exons 9 and 13 of LRP5. The wild-type or truncated LRP5 mRNAs were detected by primary PCR for most of the tumors or with nested PCR using an additional overlapping forward primer. The truncation comprised the last 93 bp of exon 9, all 227 bp of exon 10, and the first 106 bp of exon 11. (B) Nested RT-PCR of normal parathyroid tissue (*n* = 6), and parathyroid tumor cell line sHPT-1 [8] as marker (top panel). Nested RT-PCR analysis of 17 normal tissues, and the parathyroid tumor pHPT21 as marker (lower panel). (C) Immunoprecipitation followed by Western blot analysis of LRP5. Transiently expressed (HEK293T cells) LRP5 and LRP5Δ666–809 shown as size markers. sHPT-1, human parathyroid tumor cell line [8]; pHPT, pHPT tumor. The lower panel shows additional pHPT tumor samples analyzed on a 5% SDS–polyacrylamide gel, where the proteins separate more clearly. (D) A schematic structure of LRP5 is shown. The truncated mRNA contains an in-frame deletion of LRP5 between amino acids 666 and 809 (Δ666–809), encompassing the third YWTD β-propeller domain. The truncation (Δ666–809) is flanked by imperfect direct repeat sequences (horizontal arrows) with putative cryptic donor (Ac-GTG) and acceptor (AC-cT) RNA splice sites in exons 9 and 11, respectively (arrows). The Δ666–809 is between nucleotide positions 2039 and 2466 of the LRP5 mRNA (GenBank accession no. AF064548).

**Figure 2:**
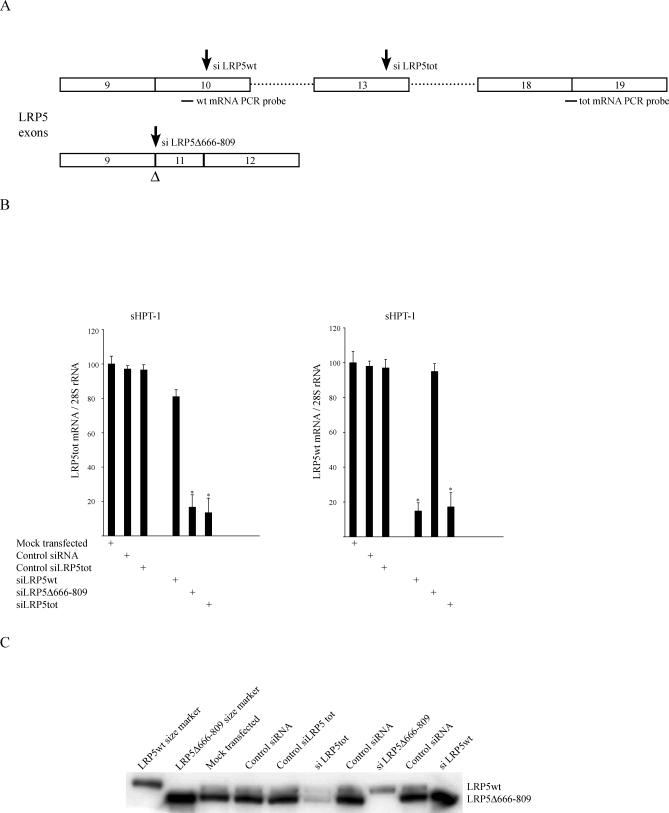
Specificity and Efficiency of siRNAs Transiently Transfected to the sHPT-1 Parathyroid Tumor Cell Line (A) Locations of siRNAs and probes for quantitative real-time PCR. tot mRNA PCR probe determines both LRP5wt and LRP5Δ666–809 transcripts. (B) Expression of LRP5wt and LRP5tot mRNA assessed by quantitative real-time PCR. (C) Immunoprecipitation and Western blot analysis of LRP5.

A shorter LRP5 transcript (LRP5Δ666–809) was detected in 32 out of 37 pHPT tumors (86%) and 20 out of 20 sHPT tumors (100%) by using RT-PCR primers located in exons 9 and 13 of LRP5 (Figure 1A). Of the analyzed parathyroid tumors (*n* = 57) which all displayed aberrant cytoplasmic/nuclear accumulation of β-catenin ([5] and unpublished data), four pHPT tumors without the LRP5Δ666–809 transcript harbored the stabilizing mutation of the β-catenin exon 3 (S37A), and one pHPT tumor had neither. Three out of the four tumors with β-catenin mutation were reported previously, and the one tumor without β-catenin mutation or the LRP5Δ666–809 transcript displayed wild-type APC expression [5]. Six normal parathyroid tissues and a panel of 17 normal tissues expressed only the normal LRP5 (LRP5wt) transcript (Figure 1B). Accordingly, a smaller LRP5 protein was detected by immunoprecipitation followed by Western blot analysis of the parathyroid tumors expressing the LRP5Δ666–809 transcript and in the human parathyroid tumor cell line sHPT-1 (Figure 1C), where *CTNNB1* is not mutated in exon 3 [8]. The expression level of the smaller form of LRP5 varied in relation to LRP5wt among the analyzed tumors. In the sHPT-1 cell line, it was expressed at a higher relative level (see also Figure 2C). Only the LRP5wt receptor was detected in HeLa cells (Figure 1C), which did not express the truncated LRP5Δ666–809 transcript (unpublished data). No apparent relationships between expression of LRP5Δ666–809 or *CTNNB1* stabilizing mutation and clinical characteristics, such as glandular weight or serum levels of parathyroid hormone or calcium, were found.

The tumor-associated shorter transcript LRP5Δ666–809 contained an in-frame deletion of 142 amino acids (Δ666–809), encompassing the third YWTD β-propeller domain [28,29] between the second and third epidermal growth factor repeats of LRP5 (Figure 1D). The deletion (Δ666–809) was flanked by imperfect direct repeat sequences with putative cryptic donor and acceptor RNA splice sites located in exons 9 and 11, respectively (Figure 1D). The 5′ putative cryptic splice site (Ac-GTG) related to the consensus sequence of major-class introns and the 3′ cryptic splice (AC-cT) related more to the minor-class [30]. The genomic sequences of exon/intron junctions and branch points in question as well as exons 10 and 11 did not reveal any abnormalities in several tumors. Also, no deletion was noted by Southern blotting of genomic DNA (unpublished data). The LRP5 internal truncation is most likely the result of cryptic splice site usage.

### The Internally Truncated LRP5 Receptor Contributes to Active β-Catenin Signaling

To investigate the functional consequences of LRP5Δ666–809 expression, if any, specific and control siRNAs were efficiently transfected [8] to the recently established unique human parathyroid tumor cell line sHPT-1. Three different siRNAs against LRP5 mRNA were used (Figure 2A): one against the LRP5wt transcript (siLRP5wt), one against the internally truncated transcript (siLRP5Δ666–809), and one recognizing both the LRP5wt and LRP5Δ666–809 transcripts (siRNAtot). siLRP5wt was directed to exon 10, which was excluded from the truncated transcript (Figure 1A); siLRP5Δ666–809 was directed against the unique sequence created at the aberrant exon junction; and siRNAtot was directed to exon 13, which was present in both LRP5 transcripts (Figures 1D and 2A). A nonsilencing siRNA (control siRNA) and a mutant siRNAtot (control siRNAtot) were also used. Specificity and silencing potential of the siRNAs were ascertained both at the mRNA and protein level (Figure 2B and 2C).

**Figure 3:**
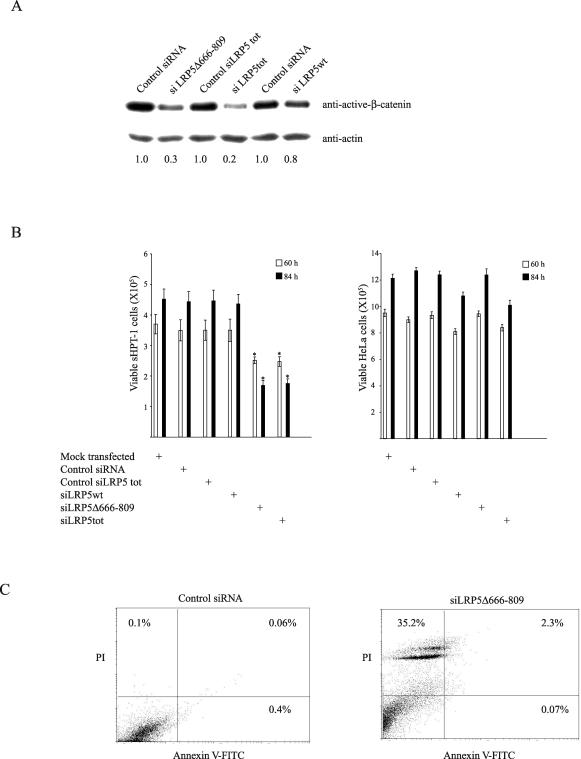
Maintained Expression of the Internally Truncated LRP5 Receptor Is Required for Accumulation of Nonphosphorylated β-Catenin and Continued Cell Growth (A) Western blot analysis of active β-catenin [25], 60 h after transfection. The β-catenin–actin signal ratio is shown. (B) Effects on sHPT-1 cell growth. HeLa cells were used as control for toxic effects. *p < 0.05. (C) Flow cytometry analysis of sHPT-1 cells at 84 h after transfection after staining with annexin V–FITC and propidium iodide. Accumulation of dead cells in the upper left quadrant; population of late apoptotic cells (upper right quadrant) and early apoptotic cells (lower right quadrant).

**Figure 4:**
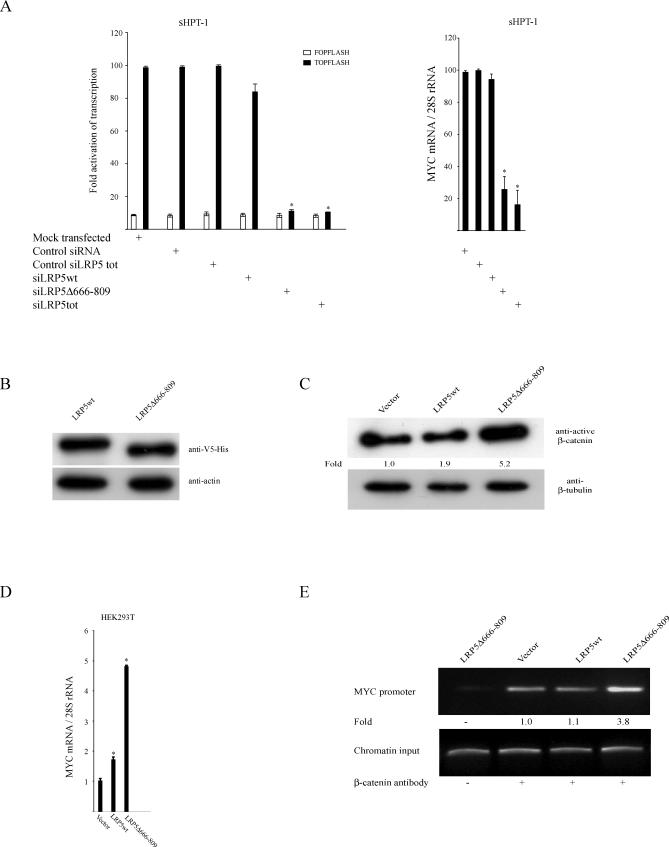
The Internally Truncated LRP5 Receptor Regulates β-Catenin–Driven Transcription (A) Transient cotransfections of FOPFLASH or TOPFLASH TCF/β-catenin reporters, the CMV-LacZ reference plasmid, and indicated siRNAs to sHPT-1 cells (left panel). FOPFLASH contains mutated binding sites for TCF factors, while TOPFLASH does not [22]. Luciferase activities were normalized to β-galactosidase activities. Effects on endogenous *MYC* expression in sHPT-1 cells (right panel). *p < 0.05. (B) Western blot analysis of V5-tagged LRP5 and LRP5Δ666–809 transiently transfected to HEK293T cells. LRP5 and LRP5Δ666–809 were expressed at similar levels. (C) Western blot analysis of cytosolic nonphosphorylated active β-catenin in HEK293T cells, 24 h after transfection. (D) Endogenous *MYC* expression in transfected HEK293T cells. *p < 0.05. (E) ChIP of the *MYC* promoter in transfected HEK293T cells. An anti-active–β-catenin monoclonal antibody was used [25].

Transfection of siLRP5Δ666–809 reduced the nonphosphorylated active β-catenin level markedly as well as decreased the growth of sHPT-1 cells. siLRP5wt showed a tendency toward a lowered β-catenin level, but no significant effect on cell growth was observed (Figure 3A and 3B). In agreement with these results, transfection of siRNA specific for both LRP5 transcripts (siLRP5tot) resulted in similar diminished β-catenin expression and cell growth as for siLRP5Δ666–809 (Figure 3A and 3B). The growth of HeLa cells, which did not express the internally truncated LRP5 receptor (Figure 1C), was not affected by siRNA transfections (Figure 3B). As shown previously for siRNA to CTNNB1 [8], sHPT-1 cells transfected with siLRP5Δ666–809 but not with control siRNA showed accumulation of dead cells as well as a smaller population of apoptotic cells 84 h after transfection (Figure 3C). Furthermore, the endogenous β-catenin activity in sHPT-1 cells (11-fold), as measured by FOPFLASH/TOPFLASH TCF/β-catenin luciferase reporter transfection, was dependent on maintained expression of LRP5Δ666–809 and not of LRP5wt (Figure 4A). Dependence on maintained expression of LRP5Δ666–809 was also observed for expression of the endogenous β-catenin target gene *MYC* [22], which is commonly overexpressed in parathyroid tumors [5], as the *MYC* mRNA level was significantly reduced in sHPT-1 parathyroid cells transfected with siLRP5Δ666–809 and siLRP5tot, but not with siLRP5wt, control siRNA, and control siLRP5tot (Figure 4A). Expression of *MYC* in sHPT-1 cells has been shown previously to depend on β-catenin [8]. Thus, maintained expression of the internally truncated LRP5 receptor in sHPT-1 cells appeared necessary for accumulation of transcriptionally active β-catenin, continued cell growth, and *MYC* expression. An effect of the internally truncated LRP5 receptor on β-catenin signaling was further substantiated by experiments in HEK293T cells. Transient expression (Figure 4B) of LRP5Δ666–809 and not of LRP5wt in these cells resulted in increased level (5-fold) of nonphosphorylated active β-catenin (Figure 4C), a 5-fold increase of endogenous *MYC* mRNA expression (Figure 4D), and enhanced association (4-fold) of β-catenin to the *MYC* promoter, as revealed by ChIP (Figure 4E).

### WNT3 Ligand and the Internally Truncated LRP5 Receptor Strongly Activate Transcription with Impaired DKK1 Inhibition

**Figure 5:**
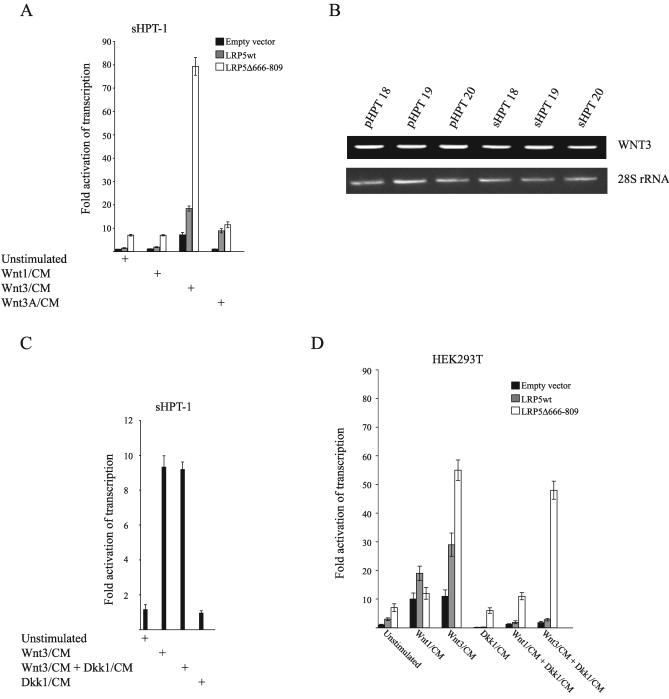
WNT3 Ligand and the Internally Truncated LRP5 Receptor Strongly Activates Transcription of the TOPFLASH TCF/β-Catenin Luciferase Reporter with Impaired DKK1 Inhibition (A) sHPT-1 cells cotransfected with TOPFLASH, LRP5wt, or LRP5Δ666–809 expression vectors and CMV-LacZ reference plasmid, followed by incubation in WNT1, WNT3, or WNT3A conditioned medium (CM). CM was from HEK293T cells transfected transiently with expression vectors for the various WNT ligands. The 11-fold (Figure 4A) endogenous β-catenin activity is set to 1 (unstimulated, empty vector). (B) Representative RT-PCR analysis of RNA from three pHPT and three sHPT tumors using primers for WNT3. No expression of WNT1 was detected by the conditions used [21]. (C) Cotransfection of TOPFLASH and CMV-LacZ reference plasmid to sHPT-1 cells. Incubation in WNT3 and DKK1 CM. (D) Cotransfection of TOPFLASH, CMV-LacZ reference plasmid, and LRP5wt or LRP5Δ666–809 expression vectors to HEK293T cells followed by incubation in WNT1, WNT3, or DKK1 CM. HEK293T cells do not express the internally truncated LRP5 receptor.

The effects of WNT ligand stimulation of the LRP5wt and LRP5Δ666–809 receptors were determined by transient cotransfection of the TOPFLASH TCF/β-catenin luciferase reporter and the LRP5 receptor expression vectors to sHPT-1 cells, followed by incubation in WNT1, WNT3, or WNT3A conditioned medium. Transfected LRP5Δ666–809 displayed activation of β-catenin driven transcription in sHPT-1 cells (9-fold), and transcription was most prominently stimulated (80-fold) in the presence of WNT3 ligand, where it was 4-fold higher than for LRP5wt (Figure 5A). In addition, only WNT3 stimulated the endogenous β-catenin activity (9-fold). WNT3A ligand stimulated transcription only in the presence of LRP5wt (10-fold), and WNT1 showed no effects. *WNT3* but not *WNT1* was expressed by RT-PCR in parathyroid tumors (Figure 5B).

Several amino acid residues in the deleted part of LRP5 (Δ666–809) were shown to be required for inhibition of β-catenin activity by the WNT antagonist DKK1 [31]. Therefore, we examined the effect of DKK1, which is expressed in parathyroid tumors (unpublished data) on WNT3-stimulated β-catenin activity in sHPT-1 cells. The presence of DKK1 in the culture medium resulted in inhibition of WNT3 activation in HEK293T control cells as expected (Figure 5D), but not in sHPT-1 cells (Figure 5C). In agreement with these results, WNT3-induced TOPFLASH transcription was inhibited by DKK1 in HEK293T cells cotransfected with LRP5wt and not with LRP5Δ666–809 (Figure 5D). Also, the activity of LRP5Δ666–809 in unstimulated cells (7-fold) was insensible to DKK1. In contrast to sHPT-1 cells (Figure 5A), HEK293T cells responded to WNT1-conditioned medium (7-fold) and was sensitive to DKK1 inhibition as expected. However, the small increase of LRP5Δ666–809 activity in the presence of WNT1 (1.7-fold) was insensible to DKK1 (Figure 5D). The impaired DKK1-mediated antagonism of β-catenin activity may contribute to overall signaling of the internally truncated LRP5 receptor.

### Inhibition of Tumor Growth in SCID Mice

**Figure 6:**
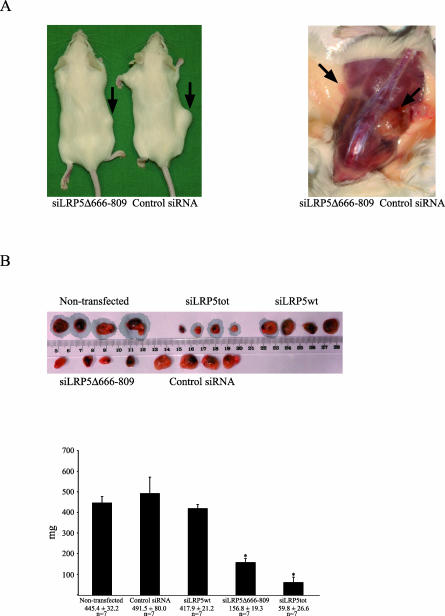
siRNAs to the Internally Truncated LRP5 Receptor Inhibit Tumor Growth in Xenografted SCID Mice (A) Injection of sHPT-1 cells pretransfected for 24 h with siLRP5Δ666–809 or control siRNA. Arrows indicate representative parathyroid tumor growth at site of transplantation. (B) Parathyroid tumors from SCID mice (*n* = 35) injected with sHPT-1 cells pretransfected for 24 h with the indicated siRNAs were excised and weighed. *p < 0.05. The animals were monitored every day and killed after 8–9 wk.

The in vivo growth properties of sHPT-1 cells were evaluated in a xenograft SCID mouse model. Significant tumor growth inhibition was observed in transplants of cells when pretransfected with siLRP5Δ666–809 or siLRP5tot, but not with siLRP5wt or control siRNA (Figure 6A and 6B). Thus, the internally truncated LRP5 receptor appeared to be required for tumor growth in this animal model. Combination of the very high siRNA transfection efficiency of sHPT-1 cells [8] and generally prolonged gene silencing in slowly dividing cells [32] are likely prerequisites for the sustained tumor growth inhibition observed 8–9 wk after transplantation.

## Discussion

β-catenin accumulation has been observed by us in all so far analyzed parathyroid tumors of primary origin and hyperplastic parathyroid glands of HPT secondary to uremia ([5] and this work), strongly suggesting dysregulated WNT/β-catenin signaling as a common pathogenic pathway for these different conditions. We reported *CTNNB1*-stabilizing mutations in a few cases (3 out of 20) of pHPT tumors, while no mutation was found in uremic secondary HPT tumors, and inactivating truncations of APC were not seen [5]. Recently, parathyroid adenomas from 97 patients with pHPT who had undergone parathyroidectomy in the United States were analyzed regarding *CTNNB1*-stabilizing mutations in exon 3 [33]. In agreement with one previous smaller study of Japanese patients [34], no mutations were identified. Taken together, these results indicated a low *CTNNB1* mutation frequency in pHPT tumors [35]. We are currently analyzing a large number of additional pHPT tumors from Swedish patients. A few additional tumors with *CTNNB1*-stabilizing mutations has been found so far, suggesting an overall mutation frequency of approximately 6% (Björklund et al., unpublished data). This frequency is slightly lower than that observed for colorectal cancers, in which approximately 10% of the tumors harbored missense mutations or interstitial deletions of exon 3 [13,14].

Several lines of experimental evidence presented here support a fundamental role of the internally truncated LRP5 receptor in regulating β-catenin–driven transcription in parathyroid tumor cells. The truncated LRP5 receptor was frequently expressed both in parathyroid tumors of primary origin (86%) and in hyperplastic parathyroid glands of HPT secondary to uremia (100%). Of all 57 analyzed parathyroid tumors with accumulated β-catenin, 52 expressed the internally truncated LRP5 receptor without *CTNNB1*-stabilizing mutations, four tumors had *CTNNB1*-stabilizing mutations but no truncated LRP5 receptor, and 1 tumor displayed neither aberration. Thus, it seems that mutation of *CTNNB1* and expression of LRP5Δ666–809 is mutually exclusive.

All reported *LRP5* missense mutations, which cause a high-bone-mass phenotype, are located before the first epidermal growth factor–like repeat in the amino terminal part of the receptor, not including the third YWTD β-propeller domain, which is absent in the truncated LRP5 receptor. Several mutations of *LRP5* known to cause osteoporosis-pseudoglioma syndrome is located within amino acids 666–809, highlighting a functional role for this part of the protein [36]. Osteoporosis-pseudoglioma mutations seem to cause varying degree of reduced Wnt or Norrin signaling by unknown mechanisms, as determined by the TOPFLASH reporter assay [37]. Clearly, the deletion of 142 amino acids in LRP5Δ666–809 and a missense mutation in the same part of the receptor have opposite effects. A missense mutation may for example interfere with binding of cell-type–specific factor(s), while a larger deletion may result in structural changes of the receptor with a different impact on cofactor interactions and signal outcome. The mechanism(s) by which the internally truncated LRP5 receptor activates β-catenin signaling in tumor cells remains to be elucidated, but may involve impaired inhibition by DKK1. The WNT3 ligand activated transient transcription more strongly in the presence of LRP5Δ666–809 than LRP5wt, possibly suggesting favored interaction of WNT3 with the internally truncated receptor. We emphasize that the conditioned medium of WNT1, WNT3, WNT3A, and DKK1 used throughout this study might contain uncharacterized signaling molecules induced by the WNTs or DKK1.

RNA splicing defects in cancer are common, but seem rarely to be caused by somatic mutations in splice sites and regulatory elements. Alternative splicing may potentially present as a diagnostic marker [38,39]. Interestingly, aberrant splicing between direct repeat sequences with potential cryptic splice sites and without mutations in selected parts of the gene has been described also for the *MDM2* oncogene in breast cancer [40]. We found no mutations or polymorphisms in the sequenced exons/branch points/intron–exon junctions of the *LRP5* gene in the analyzed tumors that potentially could explain the aberrant splicing. Mutations in other areas of the *LRP5* gene affecting splicing specificity, accuracy, or efficiency, or perhaps more likely interference of *trans*-acting factors of the splicing machinery, are other possibilities. Alternative splicing may be affected in a splicing factor concentration-dependent manner [41], and this may also apply to aberrant splicing in tumors. Identification of splicing oncogenes or splicing tumor suppressor genes is being pursued.

The results support a fundamental role of the aberrantly spliced internally truncated LRP5 receptor in activating WNT/β-catenin signaling in parathyroid tumor cells. LRP5 receptors could be attractive targets for the development of antitumor drugs that specifically inactivate the truncated receptor and leave the normal protein unaffected.

## Abbreviations

APC: adenomatosis polyposis coli
ChIP: chromatin immunoprecipitation
HPT: hyperparathyroidism
LRP5: low-density lipoprotein receptor–related protein 5
pHPT: primary hyperparathyroidism
SCID: severe combined immunodeficiency
sHPT: secondary hyperparathyroidism

## References

[pmed-0040328-b001] Marx SJ (2000). Hyperparathyroid and hypoparathyroid disorders. N Engl J Med.

[pmed-0040328-b002] Arnold A, Shattuck TM, Mallya SM, Krebs LJ, Costa J (2002). Molecular pathogenesis of primary hyperparathyroidism. J Bone Miner Res.

[pmed-0040328-b003] Åkerström G, Hellman P (2004). Primary hyperparathyroidism. Curr Opin Oncol.

[pmed-0040328-b004] Åkerström G, Hellman P, Hessman O, Segersten U, Westin G (2005). Parathyroid glands in calcium regulation and human disease. Ann N Y Acad Sci.

[pmed-0040328-b005] Björklund P, Åkerström G, Westin G (2007). Accumulation of nonphosphorylated beta-catenin and c-myc in primary and uremic secondary hyperparathyroid tumors. J Clin Endocrinol Metab.

[pmed-0040328-b006] van de Wetering M, Sancho E, Verweij C, de Lau W, Oving I (2002). The beta-catenin/TCF-4 complex imposes a crypt progenitor phenotype on colorectal cancer cells. Cell.

[pmed-0040328-b007] Meniel VS, Muncan V, Phesse TJ, Wilkins JA (2007). Myc deletion rescues Apc deficiency in the small intestine. Nature.

[pmed-0040328-b008] Björklund P, Åkerström G, Westin G (2007). Activated beta-catenin in the novel human parathyroid tumor cell line sHPT-1. Biochem Biophys Res Commun.

[pmed-0040328-b009] Imanishi Y, Hosokawa Y, Yoshimoto K, Schpani E, Mallya S (2001). Primary hyperparathyroidism caused by parathyroid-targeted overexpression of cyclin D1 in transgenic mice. J Clin Invest.

[pmed-0040328-b010] Motokura T, Bloom T, Kim HG, Juppner H, Ruderman JV (1991). A novel cyclin encoded by a bcl1-linked candidate oncogene. Nature.

[pmed-0040328-b011] Sansom OJ, Reed KR, van de Wetering M, Muncan V, Winton DJ (2005). Cyclin D1 is not an immediate target of beta-catenin following Apc loss in the intestine. J Biol Chem.

[pmed-0040328-b012] Polakis P (1999). The oncogenic activation of beta-catenin. Curr Opin Genet Dev.

[pmed-0040328-b013] Lustig B, Behrens J (2003). The Wnt signaling pathway and its role in tumor development. J Cancer Res Clin Oncol.

[pmed-0040328-b014] Giles RH, van Es JH, Clevers H (2003). Caught up in a Wnt storm: Wnt signaling in cancer. Biochim Biophys Acta.

[pmed-0040328-b015] Moon RT, Kohn AD, DeFerrari GV, Kaykas A (2004). WNT and beta-catenin signalling: diseases and therapies. Nat Rev Genet.

[pmed-0040328-b016] Tamai K, Semenov M, Kato Y, Spokony R, Liu C (2000). LDL-receptor-related proteins in Wnt signal transduction. Nature.

[pmed-0040328-b017] Pinson KI, Brennan J, Monkley S, Avery BJ, Skarnes WC (2000). An LDL-receptor-related protein mediates Wnt signalling in mice. Nature.

[pmed-0040328-b018] Wehrli M, Dougan ST, Caldwell K, O'Keefe L, Schwartz S (2000). Arrow encodes an LDL-receptor-related protein essential for Wingless signalling. Nature.

[pmed-0040328-b019] Luo W, Peterson A, Garcia BA, Coombs G, Kofahl B (2007). Protein phosphatase 1 regulates assembly and function of the beta-catenin degradation complex. EMBO J.

[pmed-0040328-b020] Mao J, Wang J, Liu B, Pan W, Farr GH (2001). Low-density lipoprotein receptor-related protein-5 binds to Axin and regulates the canonical Wnt signaling pathway. Mol Cell.

[pmed-0040328-b021] Winn RA, Marek L, Han SY, Rodriguez K, Rodriguez N (2005). Restoration of Wnt-7a expression reverses non-small cell lung cancer cellular transformation through frizzled-9-mediated growth inhibition and promotion of cell differentiation. J Biol Chem.

[pmed-0040328-b022] He TC, Sparks AB, Rago C, Hermeking H, Zawel L (1998). Identification of c-MYC as a target of the APC pathway. Science.

[pmed-0040328-b023] Knutson A, Castano E, Oelgeschlager T, Roeder RG, Westin G (2000). Downstream promoter sequences facilitate the formation of a specific transcription factor IID-promoter complex topology required for efficient transcription from the megalin/low density lipoprotein receptor-related protein 2 promoter. J Biol Chem.

[pmed-0040328-b024] Sharma M, Chuang WW, Sun Z (2002). Phosphatidylinositol 3-kinase/Akt stimulates androgen pathway through GSK3beta inhibition and nuclear beta-catenin accumulation. J Biol Chem.

[pmed-0040328-b025] van Noort M, Meeldijk J, van der Zee R, Destree O, Clevers H (2002). Wnt signaling controls the phosphorylation status of beta-catenin. J Biol Chem.

[pmed-0040328-b026] Chen H, Lin RJ, Xie W, Wilpitz D, Evans RM (1999). Regulation of hormone-induced histone hyperacetylation and gene activation via acetylation of an acetylase. Cell.

[pmed-0040328-b027] Workman P, Balmain A, Hickman JA, McNally NJ, Rohas AM (1988). UKCCCR guidelines for the welfare of animals in experimental neoplasia. Lab Anim.

[pmed-0040328-b028] Jeon H, Meng W, Takagi J, Eck MJ, Springer TA (2001). Implications for familial hypercholesterolemia from the structure of the LDL receptor YWTD-EGF domain pair. Nat Struct Biol.

[pmed-0040328-b029] Takagi J, Yang Y, Liu J, Wang J, Springer TA (2003). Complex between nidogen and laminin fragments reveals a paradigmatic beta-propeller interface. Nature.

[pmed-0040328-b030] Patel AA, Steitz JA (2003). Splicing double: Insights from the second spliceosome. Nat Rev Mol Cell Biol.

[pmed-0040328-b031] Zhang Y, Wang Y, Li X, Zhang J, Mao J (2004). The LRP5 high-bone-mass G171V mutation disrupts LRP5 interaction with Mesd. Mol Cell Biol.

[pmed-0040328-b032] Bartlett DW, Davis ME (2006). Insights into the kinetics of siRNA-mediated gene silencing from live-cell and live-animal bioluminescent imaging. Nucl Acids Res.

[pmed-0040328-b033] Costa-Guda J, Arnold A (2007). Absence of stabilizing mutations of β-catenin encoded by CTNNB1 exon 3 in a large series of sporadic parathyroid adenomas. J Clin Endocrinol Metab.

[pmed-0040328-b034] Ikeda S, Ishizaki Y, Shimizu Y, Fujimori M, Ojima Y (2002). Immunohistochemistry of cyclin D1 and β-catenin, and mutational analysis of exon 3 of β-catenin gene in parathyroid tumors. Int J Oncol.

[pmed-0040328-b035] Simonds WF (2007). Ruling out a suspect: the role of beta-catenin mutation in benign parathyroid neoplasia. J Clin Endocrinol Metab.

[pmed-0040328-b036] Levasseur R, Lacombe D, de Vernejoul MC (2005). LRP5 mutations in osteoporosis-pseudoglioma syndrome and high-bone-mass disorders. Joint Bone Spine.

[pmed-0040328-b037] Ai M, Heeger S, Bartels CF, Schelling DK (2005). Osteoporosis-Pseudoglioma Collaborative Group. Clinical and molecular findings in osteoporosis-pseudoglioma syndrome. Am J Hum Genet.

[pmed-0040328-b038] Wang Z (2003). Computational analysis and experimental validation of tumor-associated alternative RNA splicing in human cancer. Cancer Res.

[pmed-0040328-b039] Kalnina Z, Zayakin P, Silina K, Line A (2005). Alterations of pre-mRNA splicing in cancer. Genes Chrom Cancer.

[pmed-0040328-b040] Lukas J, Gao DQ, Keshmeshian M, Wen WH, Tsao-Wei D (2001). Alternative and aberrant messenger RNA splicing of the *mdm2* oncogene in invasive breast cancer. Cancer Res.

[pmed-0040328-b041] Karni R, de Stanchina E, Lowe SW, Sinha R, Mu D (2007). The gene encoding the splicing factor SF2/ASF is a proto-oncogene. Nat Struct Mol Biol.

